# COVID-19 Critical Care Simulations: An International Cross-Sectional Survey

**DOI:** 10.3389/fpubh.2021.700769

**Published:** 2021-09-21

**Authors:** Mohamad-Hani Temsah, Abdulkarim Alrabiaah, Ayman Al-Eyadhy, Fahad Al-Sohime, Abdullah Al Huzaimi, Nurah Alamro, Khalid Alhasan, Vaibhavi Upadhye, Amr Jamal, Fadi Aljamaan, Ali Alhaboob, Yaseen M. Arabi, Marc Lazarovici, Ali M. Somily, Abdulaziz M. Boker

**Affiliations:** ^1^College of Medicine, King Saud University, Riyadh, Saudi Arabia; ^2^Pediatric Intensive Care Unit, Pediatric Department, King Saud University Medical City, Riyadh, Saudi Arabia; ^3^Pediatric Infectious Disease Unit, Pediatric Department, King Saud University Medical City, Riyadh, Saudi Arabia; ^4^Clinical Skills & Simulation Center, King Saud University Medical City, Riyadh, Saudi Arabia; ^5^Division of Pediatric Cardiology, Department of Cardiac Sciences, College of Medicine, King Saud University Medical City, Riyadh, Saudi Arabia; ^6^Heart Center, King Faisal Specialist Hospital & Research Center, Riyadh, Saudi Arabia; ^7^Department of Family and Community Medicine, King Saud University Medical City, Riyadh, Saudi Arabia; ^8^Pediatric Nephrology Unit, Pediatric Department, King Saud University Medical City, Riyadh, Saudi Arabia; ^9^Clinical Lead in Simulation, Dr. Indumati Amodkar Simulation Center, Deenanath Mangeshkar Hospital and Research Center, Pune, India; ^10^Evidence-Based Health Care & Knowledge Translation Research Chair, King Saud University, Riyadh, Saudi Arabia; ^11^Critical Care Department, King Saud University Medical City, Riyadh, Saudi Arabia; ^12^National Guard Health Affairs, Riyadh, Saudi Arabia; ^13^King Saud Bin Abdulaziz University for Health Sciences, Riyadh, Saudi Arabia; ^14^King Abdullah International Medical Research Center, Riyadh, Saudi Arabia; ^15^Ludwig-Maximilians-University, Munich University Hospital, Munich, Germany; ^16^Department of Pathology and Laboratory Medicine, King Saud University Medical City, Riyadh, Saudi Arabia; ^17^Anesthesia and Critical Care Department, King Abdulaziz University Hospital, Faculty of Medicine, King Abdulaziz University, Jeddah, Saudi Arabia; ^18^Clinical Skills and Simulation Centre, King Abdulaziz University, Jeddah, Saudi Arabia

**Keywords:** COVID-19, simulations, healthcare workers, healthcare preparedness, international survey

## Abstract

**Objective:** To describe the utility and patterns of COVID-19 simulation scenarios across different international healthcare centers.

**Methods:** This is a cross-sectional, international survey for multiple simulation centers team members, including team-leaders and healthcare workers (HCWs), based on each center's debriefing reports from 30 countries in all WHO regions. The main outcome measures were the COVID-19 simulations characteristics, facilitators, obstacles, and challenges encountered during the simulation sessions.

**Results:** Invitation was sent to 343 simulation team leaders and multidisciplinary HCWs who responded; 121 completed the survey. The frequency of simulation sessions was monthly (27.1%), weekly (24.8%), twice weekly (19.8%), or daily (21.5%). Regarding the themes of the simulation sessions, they were COVID-19 patient arrival to ER (69.4%), COVID-19 patient intubation due to respiratory failure (66.1%), COVID-19 patient requiring CPR (53.7%), COVID-19 transport inside the hospital (53.7%), COVID-19 elective intubation in OR (37.2%), or Delivery of COVID-19 mother and neonatal care (19%). Among participants, 55.6% reported the team's full engagement in the simulation sessions. The average session length was 30–60 min. The debriefing process was conducted by the ICU facilitator in (51%) of the sessions followed by simulation staff in 41% of the sessions. A total of 80% reported significant improvement in clinical preparedness after simulation sessions, and 70% were satisfied with the COVID-19 sessions. Most perceived issues reported were related to infection control measures, followed by team dynamics, logistics, and patient transport issues.

**Conclusion:** Simulation centers team leaders and HCWs reported positive feedback on COVID-19 simulation sessions with multidisciplinary personnel involvement. These drills are a valuable tool for rehearsing safe dynamics on the frontline of COVID-19. More research on COVID-19 simulation outcomes is warranted; to explore variable factors for each country and healthcare system.

## Introduction

The COVID-19 crisis started early January 2020 when the World Health Organization (WHO) reported a cluster of pneumonia cases in Wuhan, China (1). Soon the disease was declared a pandemic by the WHO in March 2020, which urged healthcare systems to initiate rapid training for their healthcare workers (HCWs) to cope with this rapidly evolving pandemic.

COVID19 has been labeled by the WHO as a novel infection that spreads quickly across the globe and is associated with high fatality and transmission rates. Its widespread mode of transmission mandated broad infection control precautions empirically. Adding to that the flooding incidence rates in some countries, all together mandated conduction of simulation programs and drills of different scenarios in order to boost the preparedness standards of the healthcare institutions. Recently, with the Delta variant, COVID-19 continued to spread even more aggressively than the original strain around 4 million cases worldwide reported in 1 week to the WHO, and the number of cases is expected to exceed 200 million in August 2021 (2).

COVID-19 with the disruption it caused to humankind at various levels, medical economic and even educational can be classified as a biological threat or disaster. Therefore preparedness of the healthcare system is of paramount importance as early as possible, that includes training of the medical staff for the different infection control measures at different scenarios, environmental contamination, medical management„ preparing senior staff to deal with ethical issues related to medical decisions expected to be encountered during the COVDI-19 crisis either at individual level or institution one, and psychosocial preparedness of the medical and adminstrative staff, this has been recommended by The European Society of Intensive Care Medicine's Task Force for Intensive Care Unit (3). All these aspects could be assets to healthcare administration and policymakers.

Model simulation research suggests that COVID-19 might become a seasonal disease and persist for more extended periods, hence the importance of ongoing training for the HCWs (4, 5). Mathematical modeling to better understand the disease dynamics in order to control its rapidly spreading infectious nature highlights the importance of infection control measures in order to control the basic reproduction number R_0_ and maintain the endemic equilibrium (6), keeping in mind that local and global dynamics are determined by the threshold parameters R0 and R1 (7).

Literature highly supports prompt establishment of highly effective simulation programs for such evolving pandemics. During the 2003 severe acute respiratory syndrome (SARS) crisis, Abrahamson et al. proposed and tested a novel protocol for cardiac arrest in a patient with SARS, the protocol was promptly and effectively instituted by teamwork training in high-fidelity simulation drills (8). A Cochrane review concluded that face-to-face training in PPE use might reduce errors better than video- or folder-based training (9). Previous models that addressed intensive care units and hospital preparations for an influenza epidemic or mass disaster suggested enforcing communication, coordination, and collaboration between the ICU and key interface departments (10), and emphasized the beneficial effects of proper training, including Simulation-Based Education (SBE), during various coronavirus outbreaks on HCWs' mental health and well-being (11–13). Model simulation research suggests that COVID-19 might become a seasonal disease and persist for more extended periods, hence the importance of ongoing training for the HCWs (4, 5). Another mathematical modeling to better understand the disease dynamics to control the rapidly spreading infectious disease demonstrates the importance of infection control measures to control the basic reproduction number R_0_ and maintain the endemic equilibrium (6), keeping in mind that local and global dynamics are determined by the threshold parameters R0 and R1, and that dynamics of any proposed model still deserve more work to be done in the future (4–7, 14).

Therefore (SBE), is an important training tool that could especially be used to test and boost preparedness for global pandemics or natural disasters or man-made mass casualties' incidents (15). Even before this worldwide pandemic, access to, utilization of, and willingness to engage in healthcare simulation was variable among health care workers and systems (16). Therefore, sharing the experience from simulation centers worldwide potentially would benefit sites scarce of them during the current pandemic. Additionally, could help healthcare providers and policymakers expedite solutions to rectify commonly encountered errors dealing with COVID-19 patients and prevent potential outbreaks, especially in healthcare institutions associated ones which have disastrous consequences on the healthcare system. Overall (SBE), would improve patients' outcomes, maintain healthcare system integrity (17).

We conducted this international survey to describe the characteristics of COVID-19 simulation sessions (drills) among international healthcare centers where COVID-19 simulations were conducted, explore the facilitators and barriers to COVID-19 simulations, explore participants' feedback on COVID-19 simulations and evaluate the participants' Perception of infection control measures adherence.

## Method

Data collection was conducted using an online survey on Survey Monkey^®^. The research team developed the questionnaire following multidisciplinary team meeting and brainstorming of simulation experts based on their expertise in the medical simulation setting and the debriefing reports' from three simulation centers. The content validity of the questionnaire was tested with another four simulation lab experts.

Site recruitment: Hospitals equipped with simulation centers across 30 countries of the WHO regions were contacted wherever COVID-19 simulation drills were performed (18). Participants were invited during the survey period (14 April 2020-27 May 2020) either by convenient sampling (link sent by direct contact to the international simulation center leaders), or sharing of the survey through healthcare, professional social media groups on WhatsApp or Telegraph, or via emails to simulation center societies (listed at https://www.ssih.org/Home/SIM-Center-Directory).

To improve the quality of reporting, our study followed the Strengthening the Reporting of Observational Studies in Epidemiology (STROBE) guidelines (19).

### Study Population

The inclusion criteria comprised simulation team leaders and HCWs who participated in drills performed specifically for COVID-19 scenarios and, followed by a debriefing in the recruited sites. The surveyed HCWs include physicians, nurses, respiratory therapist, or simulation technologists who attended the meeting. Simulation sites or participants without documented debriefings were excluded.

Enrollment and consent: Before participation, the purpose of the study was explained in English at the beginning of the electronic survey. The respondent was given the opportunity to ask questions via a dedicated email address for the study. The institutional review board (IRB) at King Saud University approved the study (approval # 20/0273/IRB) and waived the signed consent since the evaluation presented no more than minimal risk to subjects and involved no procedures for which written consent is usually required outside the study context. To maximize confidentiality, personal identifiers were not required.

Data collection was conducted using the survey items. Following a literature review of previous simulation studies' questionnaires, the survey items were adopted and modified to answer the current research questions (8). The participants were asked about characteristics of COVID-19 simulation drills at their site, facilitators and barriers to the sessions, and feedback on them.

### Patient and Public Involvement

No patients were involved in this research.

### Data Management and Analysis

Data collection was completed based on electronic surveys utilizing the questionnaire. The data from the questionnaires were transferred into an Excel database. The data was then cleaned and analyzed using SPSS.

### Statistical Data Analysis

Means and standard deviations were used to describe the continuous variables and the frequencies and percentages for the categorically measured variables. A multiple response dichotomy analysis was used to describe the questions that allowed the selection of more than an option with a higher rank indicating higher importance. Statistical analysis was done using Statistical Package for the Social Sciences (SPSS) version 21 for windows 8.1 “IBM Corp. Released 2012. IBM SPSS Statistics for Windows, Version 21.0. Armonk, NY: IBM Corp.” (20). Microsoft Excel V16.43.1^®^ was used for the creation of figures and depictions.

## Results

From the 343 participants who were contacted, only 121 participants completed the survey and were included in the analysis.

### Demographic of the Respondents

Most respondents were female (62.8%), and about 50% of the participants” age ranged between 25-44 years (Table 1). More than half of the respondents (56.2%) were physicians, while the rest were from other allied healthcare specialties. Most (76%) of the respondents were HCWs while only 24% were simulation teams' leaders/organizers. The geographical distribution of respondents' centers were as of the following: 41.3% from the East Mediterranean (EMRO) countries region, 25.6% from the Southeast Asian countries (SERO) region, 12.4% from Europe, and the remainder from other regions (Supplementary Figures 1A,B) (21).

**Table 1:** Descriptive analysis of participants' sociodemographic and professional characteristics.

	**Frequency (*N*)**	**Percentage (%)**
**Sex**		
Female	76	62.8
Male	45	37.2
Age (years)	121	
18–24 years	16	13.2
25–34 years	29	24
35–44 years	36	29.8
45–54 years	40	33.1
**Clinical Role**		
Physician	68	56.2
Nurse and Respiratory therapist	53	43.8
**Country of practice**		
Europe (EURO) Region	15	12.4
East Mediterranean (EMRO) Region	50	41.3
Africa (AFRO) Region	7	5.8
Region of the Americas (PAHO)	8	6.6
South-East Asia (SERO) Region	31	25.6
Western Pacific Region (WPRO) Region	10	8.3
**Role in the simulation experience:**		
Simulation Team Leader/Organizer	29	24
Healthcare provider attending in the simulation	92	76

*N = 121*.

### The Nature of COVID-19 Simulations or Drills Based on Respondents

The nature and composition of COVID-19 simulations are shown in Table 2. The institutions varied in the number of conducted drills, most of the centers conducted more than ten sessions (28.9%), while (37.4%) conducted 2–4 sessions.

**Table 2:** Description of COVID 19 simulation sessions (Drills) based on respondents (*N* = 121).

	**Frequency (*N*)**	**Percentage (%)**
**How many simulations are done for COVID-19 patients in your setting?**		
One session	22	18.2
2–4 sessions	45	37.2
5–10 sessions	19	15.7
>10 sessions	35	28.9
**How often are simulations done for COVID-19 patients in your setting?**		
Once only per month	33	27.3
One per week	30	24.8
Two per week	24	19.8
Almost daily	26	21.5
Other (please specify)- sporadic	8	6.6
**Approximately what is the number of HCWs who participated in all your COVID-19 simulations until now?**		
<10 persons	21	17.4
10–50 persons	41	33.9
51–100 persons	11	9.1
More than 100 persons	38	31.4
Not counted	10	8.3
**The average time of the session (including debriefing)**		
Up to 30 min	30	27.5
30–60 min	44	40.4
more than 1 h	35	32.1
**Which department conducted the simulation in your hospital? (Multiple responses apply)**		
Simulation center	67	55.4
ICU team	51	42.1
ER team	35	28.9
OR team	18	14.9
Other Teams	25	12.8
**Staff present during sessions (Multiple responses apply)**		
Physicians	101	83.5
Nurses	92	76
Respiratory therapy staff	57	47.1
Paramedics	26	21.5
Infection control staff	56	46.3
Simulation center staff	53	43.8
Unit leaders	53	43.8
Other staff	14	11.6
**Who was briefed before the sessions (Multiple responses apply)**		
Nobody	28	24.1
Key managers	49	42.2
Site managers	34	29.3
Other personnel	35	29.7
**Who conducted the debriefing after the sessions (Choose all that apply)**		
ER COVID-19 simulation facilitator	39	32.2
ICU COVID-19 simulation facilitator	62	51.2
Infection Control officer attending the session	31	25.6
Staff from the Simulation Center	50	41.3
Other personnel	13	10.7
**Relation of simulation session to training courses**		
Before training	37	30.8
After training	23	19.2
Unrelated to training	33	27.5
**Was the simulation recorded (stat photo or videos)**		
Yes, for debriefing purposes	42	34.7
Yes, for educational purposes	53	43.8
No	46	38
**Nature of COVID-19 Scenarios for simulation?**		
COVID-19 patient arrival to ER	84	69.4
COVID-19 patient intubation due to respiratory failure	80	66.1
COVID-19 patient requiring CPR	65	53.7
COVID-19 transport inside the hospital	65	53.7
COVID-19 elective intubation in OR	45	37.2
Delivery of COVID-19 mother and neonatal care	23	19
Other (please specify)	16	13.2

The frequency of the sessions was distributed almost evenly as follows: monthly (27.1%), weekly (24.8%), twice weekly (19.8%), and daily (21.5%). There personnel attendance of the sessions varied across the centers; one-third reported 10–50, another third reported more than 100 people, while 17.4% reported <10 participants only.

The average length of each session was 30–60 min in 40% of the cases, while those lasting <30 min or more than an hour, each was in one-third of the centers. Regarding the setting of the sessions, most of the sessions were conducted in and by staff from the simulation center, followed by the ICU and emergency room (Table 2).

### Briefing and Scenario Selection

In 25% of the session, no one of the participants was briefed before the session, while 42.2% reported that key managers of the sessions only and 29.3% of the site managers were present in the preparatory briefing.

Concerning the debriefing process, it was conducted mainly by the ICU facilitator in (51%) of the sessions followed by simulation staff (41%) of the sessions (Table 2).

The simulation sessions were followed by training courses in (30.8%), preceded by them in (19.2%), and unrelated to them in 27.5% of the cases. Most simulation sessions were recorded for debriefing or education purposes. The simulation drills were of different clinical scenarios pointing to the awareness of the centers of the potential scenarios those patients might be involved in, especially the nature of the disease being acute and respiratory (e.g., elective intubation, transport, etc.) were used, as detailed in Table 2.

### Respondents' Perceptions About Their Institutional COVID-19 Simulations

Table 3 illustrates the respondents' perceptions and attitudes about their institutions' COVID-19 simulation sessions. Five percentage of the respondents perceived that the sessions minimally improved their centers' preparedness to COVID-19 crisis. In contrast, 80% reported “a lot of” or “a great deal of” clinical preparedness improvement after the sessions.

**Table 3:** Participants' perceptions and attitudes about their institutions' COVID-19 simulation sessions.

	**Frequency (*N*)**	**Percentage (%)**
**How much did the COVID-19 simulation improve your own healthcare preparedness?**		
Minimal	6	5
A moderate amount	20	16.5
A lot	47	38.8
To a great deal	48	39.7
**How engaged were the participating staff with the simulation**		
Minimal engagement	3	2.4
A little engaged	1	0.8
Moderately engaged	39	31
Highly engaged	13	10.3
Fully engaged	70	55.6
**Overall, how would you rate the interactions of the simulation audience during the practice drill?**		
Fair	2	1.7
Good	27	22.3
Very Good	56	46.3
Excellent	36	29.8
**How well did COVID-19 simulations meet your expectations?**		
Much worse than expected	1	0.8
Worse than expected	1	0.8
About the expected	32	26.4
Better than expected	53	43.8
Much better than expected	34	28.1
**Do you think the COVID-19 drill you conducted was too long, too short, or about right?**		
Much too short	1	0.8
Too short	4	3.3
About right length	108	89.3
Too Long	8	6.6
**Did you face any risky encounters during your COVID-19 simulations?**		
Yes (please specify)	14	11.6
No	107	88.4

*N = 121*.

Most of the HCWs (55.6%) reported full engagement by themselves and their peers with the sessions, 10.3% had a high engagement, and 31% reported moderate one. Regarding the respondents' overall rating of the sessions (43.8%), felt sessions were better or much better (28.1%) than expected. The majority of respondents felt sessions were of appropriate duration. Interestingly (11.6%) indicated that they encountered what they considered risky practices during the COVID-19 simulation session, such as inadequate social distancing during the drill or insufficient surface sanitizing.

Supplementary Figure 3 shows respondents' responses regarding the time required to put on PPE during COVID emergency simulation sessions; 9.2% required 1 min or less, 23.9% 2 min, 22% 3 min, while the rest needed longer durations.

### Obstacles Perceived During COVID-19 Simulations

The HCWs were asked to rate the importance and frequency of challenges or problems encountered during the simulations of COVID-19 sessions (Table 4). They fell into four categories:

- Infection Control-Related Issues: The top issue faced were the high number of participants attending the drill, followed by donning and doffing the personal protective equipment (PPE), failure of attaching the HME viral filter during Ambu-bag ventilation which is a hazardous practice when approaching COVID-19 patients, and related to that “not clamping” the endotracheal tube during the disconnect period for suctioning to prevent viral spread to the surrounding. Other issues are described in Table 4.
- Team Dynamics-Related Issues: The top three issues encountered were challenges and unfamiliarity of communication while wearing the face mask and face shields, followed by challenges of applying advanced life support algorithms to COVID-19 patients, especially concerning intubation and handling airways, and the lack of orientation among some participants of their specific roles when dealing with COVID-19 patients.
- Logistics-Related Issues: The top issues were such as obtaining portable chest x-rays, ventilators and related equipment timely fashion.
- Patient Transport-Related Issues: The top issue was obstacles of communication with the receiving team in relation to the transport process, infection precautions not applied properly during transport process; other issues are shown in Table 4.

**Table 4:** Descriptive analysis of issues and problems perceived during COVID-19 simulation.

	**Rank**	**Mean (SD)** ^*^
**Infection control issues encountered during the covid-19 simulation**		
The high number of HCWs during the simulation around the patient that could be minimized.	1	2.54 (1.17)
Problems with donning PPEs (example: wrong sequence, improperly worn)	2	2.13 (1.10)
Problems with doffing PPEs (example: removed outside the patient room, wrong sequence, improperly disposal in the designated waste bag)	3	2.10 (1.05)
Team members frequently touching their faces while PPE is on	4	2.04 (1.01)
Lack of participating staff awareness regarding healthcare facility policy and procedures for suspected COVID-19 patients as presented during the drill	5	2.01 (1.08)
Wrong isolation measures for the patient (example: doing aerosolization procedure without airborne precautions).	6	1.96 (1.10)
Failure to disinfect hands appropriately.	7	1.93 (1)
Wrong N95 size was used or improperly placed.	8	1.87 (1)
Not wearing goggles or face shields for aerosolization-producing procedures (such as endotracheal intubation).	9	1.81 (0.99)
**TEAM DYNAMICS-RELATED ISSUES ENCOUNTERED DURING COVID-19 SIMULATION**		
Communicating through the mask & shield was challenging	1	2.87 (1.26)
Difficulties/errors in advanced life support (ACLS) for COVID	2	2.16 (1.08)
Lack of awareness among HCWs about their specific role in the COVID-19 team	3	2.16 (0.92)
Difficulties/errors in basic life support (BLS) for COVID-19 patient	4	2.13 (1.11)
Lack of awareness about their healthcare facility policy and procedures for suspected COVID-19 patients as presented in the drill	5	2.12 (1.03)
Wrong team member composition (Lack of some specialties)	6	2.04 (.98)
**LOGISTICS-RELATED ISSUES ENCOUNTERED DURING COVID-19 SIMULATION**		
Logistic support problems (such as: getting portable CXR, utilization of portable ventilator)	1	2.19 (1.14)
Not attaching the viral filter (HME filter) to the BMV during manual breath Ambu-bagging	2	2.06 (1.14)
The endotracheal tube (ETT) is not clamped during disconnection from the ventilator to prevent viral spread.	3	1.83 (1.12)
Difficulty in operating the mechanical ventilator.	4	1.63 (0.83)
**THE TRANSPORT-RELATED ISSUES ENCOUNTERED DURING COVID-19 SIMULATION**		
Difficulties in communicating with the other receiving team inside the hospital.	1	2.10 (1.08)
Transport with plastic sheet wrap is not done.	2	2.07 (1.3)
Lack of transport policy.	3	1.87 (1.10)
The transport plan inside the hospital was not clear, including a predetermined route for COVID-19 suspected patients	4	1.79 (1.05)

* *(1, Never; 2, occasionally; 3, sometimes; 4, frequently; and 5, almost always)*.

### HCWs' Perceived Challenges and Potential Facilitators for Successful COVID-19 Simulations

Respondents were asked to report the challenges and difficulties that stressed their simulation experience and facilitating factors that enhanced it. They are displayed in details in Table 5.

**Table 5:** Respondents perceived challenges and potential facilitators for successful COVID-19 simulations.

	**Frequency (*N*)**	**Percentage (%)**
**CHALLENGES FACED DURING THE COVID-19 SIMULATION**		
Risk of crowded HCWs during the simulation session while social distancing is advised	63	52.5
Depletion of PPEs while there is a limited stock during the pandemic	63	52.5
A high number of hospital units asking for COVID-19 simulation drills beyond institution resources	35	29.2
Busy Infection Control colleagues unable to attend your drill	27	22.5
Busy simulation center staff unable to attend your drills	23	19.2
Lack of administrative support for COVID-19 simulation	13	10.8
Other challenges	9	7.5
**WHAT MADE YOUR SIMULATION/ DEBRIEFING OUTCOME EFFICIENT**		
Scheduled drill so the HCWs could get ready	64	53.3
Both sudden or scheduled drills showed important findings for debriefing	45	37.5
Making the drill sudden without previous knowledge of the HCWs	28	23.3

*N = 121*.

The most common factor was non-compliance with social distancing (52.5%), followed by limited resources (29.2%), followed by limitation of attendance of key personnel during the simulation sessions like infection control officer or simulation trainer or administrative representative. 52.5% of the respondents reported their concerns of potential PPE depletion during the COVID-19 pandemic.

Regarding the facilitators of successful simulation, 53.3% of the respondents felt that a scheduled drill is a mediator of success. On the other hand, 23.3% felt unscheduled drills are one of the best facilitator for success, while 37.5% felt that feedback and debriefing process are much more important to improve future experience than the timing of the sessions.

## Discussion

Safety and its quality insurance in the medical field has been progressing slowly for long time compared to other scientific or career fields, only until the last 20 years when the policy makers and medical administratives draw more attention to that trajectory of the health care system performance, simulation has been one of the key elements in this quest. The most common way of demonstrating and learning about safety culture is simulation training (22).

Although today no other high-risk professional training exist without simulation, the progress in healthcare simulation has not been as rapid as initially predicted (22). This might be due to several factors, most prominently, while most other professions implementing a wide use of simulation are heavily based on technology, healthcare systems depend mainly on humans, which makes simulation challenging to implement (23, 24). Simulation training is a valuable method for highlighting latent safety threats in healthcare systems, find solutions, and troubleshooting them (25–27). The use of simulation in healthcare training for natural disasters, bomb threats, or infectious pandemics like the COVID-19 crisis is of utmost importance in this regard (17, 28).

Owing to its superiority in achieving its goal and the ability to detect latent safety threats, *Hands- on* simulation has been more widely applied recently, still its application varied globally (26, 29).

Therefore due to its scarcity and limited availability to certain countries, collaborative multicenter research offers many advantages over single-center simulation, including larger sample sizes for results generalizability, the ability to share findings amongst collaborative simulation sites, and networking capabilities (30). The response rates we achieved in our study from various international regions was a bit frustrating, but this might be explained by the small numbers of COVID-19 simulations centers globally, adding to that our survey was done during the peak months of the pandemic when all the healthcare system and its HCWs were super busy in patient care.

According to our study, about 45% of the surveyed centers conducted at least 5 drills till May 2020, and in 40% of the cases, it was conducted twice weekly or even daily since the announcement of the pandemic, that reflects a high alert level since the announcement of the pandemic. Some simulation centers reported conducting more than 100 drills, both in clinical areas and in their labs (31). When centralized regional COVID-19 simulations were implemented, the number of simulations was rapidly increased to more than 400 acute care simulation session requests across Alberta's broad geographical zones within 5 weeks (32).

The sessions involved at least 50 personnel in total and reached more than 100 in 30% of the surveyed institutes; in at least 75% of the surveyed institutes, the sessions lasted at least 30 min and more than an hour in about 10%, those facts reflect the effective and highly resourced planning of those sessions in many centers withing few months of the pandemic.

Almost 90% of the drills were conducted either in simulation lab, ER, or ICU setting and by their staff, pointing that those institutes took COVID-19 drills at high consideration level and conducted the drills at the portal of entry of those patients or in areas where handling them might be challenging and create chaos especially when dealing with airways and what challenges it creates from infection control point of view necessitating a high level of preparedness.

Our results have shown that the drills were well-conducted most of the time, as reflected by staff satisfaction and feeling of improvement post drills, pointing to the efficacy of those drills to face the pandemic and reassure the anxious staff. Fear can hamper performance and may hinder learning. It is essential that HCWs feel safe while learning, a simulation scenario provides a safe environment to learn and practice a broad range of possible hazardous situations (33). The feeling of fear regarding caring for infectious patients requiring strict isolation was damped after the participants had completed the simulation courses, as evidence by the majority of the surveyed HCW who reported that their preparedness improved “a lot” or “a great deal.” Based on these findings, simulation-based learning positively imparted confidence, capability, and knowledge to HCWs. When they feel safe, they will be able to deliver better care to the patients and protect themselves. Literature varied regarding the perception of HCWs readiness post simulation-based training. Prescott and Garside reported that all the participants in their study felt well-prepared for the assigned tasks after simulation-based training (34). However, another study found that fewer participants felt prepared for tasks for which they had received simulation-based training (35). Participants in the study by Khan and Kiani believed from the beginning that their colleagues who did not attend the course will be less prepared to handle COVID-19 patients (36). In a COVID-19 simulation assessment, Cheung et al. found significant improvement in all domains of personal strengths among 1,415 hospital staff members (31).

Our findings indicate variability in the video recordings of the COIVD-19 drills. For the debriefing or educational purposes, video recordings were also applied to the COVID-19 simulation. Ahmed et al. reported using video recordings of the session that were then played before the subsequent COVID-19 simulation learning session as a pre-briefing (37). Mistakes in performance could be systematically identified and discussed among the participants.

The most frequent and highly ranked challenges in terms of infection control and team dynamics-related issues faced during the COVID-19 simulations were reflective of reality clinical practice challenges. For example, HCWs' crowdedness during the drills and lack of compliance with infection control practices in this study have been reported as leading causes of HCW-related infection in many healthcare systems' COVID-19 outbreaks (38, 39). The same findings were noted by Erich Hanel et al. in their surveys (40).

Challenges with providing basic life support and advanced cardiac life support to COVID-19 patients have another challenging issue during simulation. A recent nationwide Canadian study identified many similar findings and challenges across urban and rural health care settings and addressed simulation-based education to achieve system-based learning in that regard (32).

The reported challenges during clinical based simulation provide a rich source for individual, team, and institutional gap analysis and work as a need assessment tool. These challenges serve as a stimulus for rapid cycle deliberate practice, resulting in increased preparedness. Such an approach has been successfully used in many healthcare systems to prepare hospitals or a particular section of healthcare facilities during the COVID-19 crisis worldwide (41, 42).

Previous studies have demonstrated the utility of clinical based simulation to advance healthcare provider skills and aid in developing protocols and procedures (43, 44). However, the use of simulation under the constraints inflicted by a pandemic needs further studies. Indeed, the COVID-19 pandemic poses new challenges to their execution. These challenges include limited time, personnel, and personal protective equipment (PPE). Although simulation lab capacities are overwhelmed internationally during this pandemic, only 29% of HCWs considered this to be a challenge. Given these legitimate concerns, it is essential that simulation continues to go on. Despite the pandemic and to overcome these challenges, formats of combined clinical based/hands-on and virtual video-based simulation might offer the most protected, safe environment. Virtual solutions were applied frequently among HCWs during the pandemic, as incorporating such tools for the simulation could boost the resilience of the healthcare systems during medical emergencies (45–47). This combination model also allows simulation leaders to identify and modify site-specific latent safety threats, which are system-based threats to patient safety that were not previously recognized (26). Moreover, this method offers a means of rapid knowledge dissemination, resource and time saving, while allowing for social distancing, eliminating geography as a limitation to education delivery, and allowing preparation of HCWs for clinical based/hands-on simulation training (26, 48). Of note, a recent COVID-19 simulation study found no significant differences between clinical based/hands-on and lab-based simulations for all domains of personal strengths that were assessed among their candidates (31).

The dissemination of reported simulation from international sites could improve HCWs' preparedness and behavioral response in the presence of a global crisis through reliable literature and social media advertisements (49). This could alleviate some of the stress and anxiety among the HCWs, especially in light of the new SARS-CoV-2 variants and misinformation about the COVID-19 vaccines due to unreliable sources of information about the pandemic (11, 12, 50–53). Healthcare authorities should promote coping strategies and resilience, with special attention to the frontline and acute care HCWs, with the provision of adequate protective supplies and organization of simulation and support services (54, 55). This is especially true as a recent systematic review showed a lack of both quantitative and qualitative evidence from studies during infectious disease outbreaks that could inform healthcare leaders on interventions beneficial to the resilience and mental health of frontline HCWs (56).

## Study Limitations

We included centers with simulation centers to survey institutions trained already in simulation drills; this limits our study results as this pandemic is a global pandemic affecting any healthcare institution. The cross-sectional study design does not allow inferences from the results, and the causality remains uncertain. The small sample size in our study precludes referential statistics from reaching statistically significant numbers.

Our questionnaire was available online worldwide, and we got variable replies from many countries representing different regions of the globe. Some regions were represented more heavily, while we got few replies from others. South America was not represented in our sample, and in Europe, we received data only from Spain, the U.K., Italy, Germany, and Russia. Given the diversity of European healthcare systems and the varying degrees of impact of the COVID-19 pandemic in different European countries, the data may not represent the full bandwidth of the reality in European healthcare. Another region with representation that might lead to selection bias is Africa—our responses here are from Egypt, Morocco, and South Africa. While these countries reflect a certain diversity of healthcare systems on the continent, we would not assume that our data represent the whole African reality.

While this is among the first international studies to explore the effects of several factors on COVID-19 simulations across all WHO regions, the number of responding simulation centers was relatively low. However, the information shared from all centers and HCWs was abundant. This study is subject to the limitations of cross-sectional surveys, including sampling, response, and recall biases. While we attempted to reach out to as many simulation centers as possible on the international level, there were many logistical difficulties in getting replies from several places, probably reflecting the busy and overwhelmed healthcare system during this COVID-19 crisis (18). While our study aimed to explore lessons learned from COVID-19 in international simulations, to tailor this more, we suggest conducting similar studies in the future at national, regional, or continental levels. That would explore more unique factors for each particular country and healthcare system.

## Conclusion

Globally, healthcare workers reported positive feedback from the COVID-19 simulations conducted *in situ* or in simulation labs. The presence of infection control personnel in the multidisciplinary team during the drill is warranted to highlight any latent hazards that could be addressed in advance. *In situ* simulation provides a valuable tool to rehearse the safe dynamics of HCWs on the frontline of COVID-19. More research on COVID-19 simulation outcomes is warranted to explore variable factors for each country and healthcare system.

## Data Availability Statement

The original contributions presented in the study are included in the article/Supplementary Material, further inquiries can be directed to the corresponding author.

## Author Contributions

M-HT planned and conducted the study and is responsible for the overall content as guarantor. AAlr, AA-E, and FA-S analyzed the data and wrote the results and discussion. AAl and NA contributions in the method and the manuscript drafting and finalization. VU surveyed simulation centers. FA, KA, AAlh, and YA contributions in the manuscript drafting and finalization. ML and AB contributions in the study design, data collection, and manuscript drafting and finalization. AS and AJ finalized and submitted the paper. All authors contributed to the article and approved the submitted version.

## Funding

Deanship of Scientific Research, King Saud University for funding through Vice Deanship of Scientific Research Chairs. The sponsor had no influence on the study design or the reporting of the results.

## Conflict of Interest

The authors declare that the research was conducted in the absence of any commercial or financial relationships that could be construed as a potential conflict of interest.

## Publisher's Note

All claims expressed in this article are solely those of the authors and do not necessarily represent those of their affiliated organizations, or those of the publisher, the editors and the reviewers. Any product that may be evaluated in this article, or claim that may be made by its manufacturer, is not guaranteed or endorsed by the publisher.
