# Supplementary figures and images for: COVID-19 Critical Care Simulations: An International Cross-Sectional Survey
Source: Front Public Health. 2021 Sep 21;9:700769. doi: 10.3389/fpubh.2021.700769 (PMC8500233; doi:10.3389/fpubh.2021.700769)

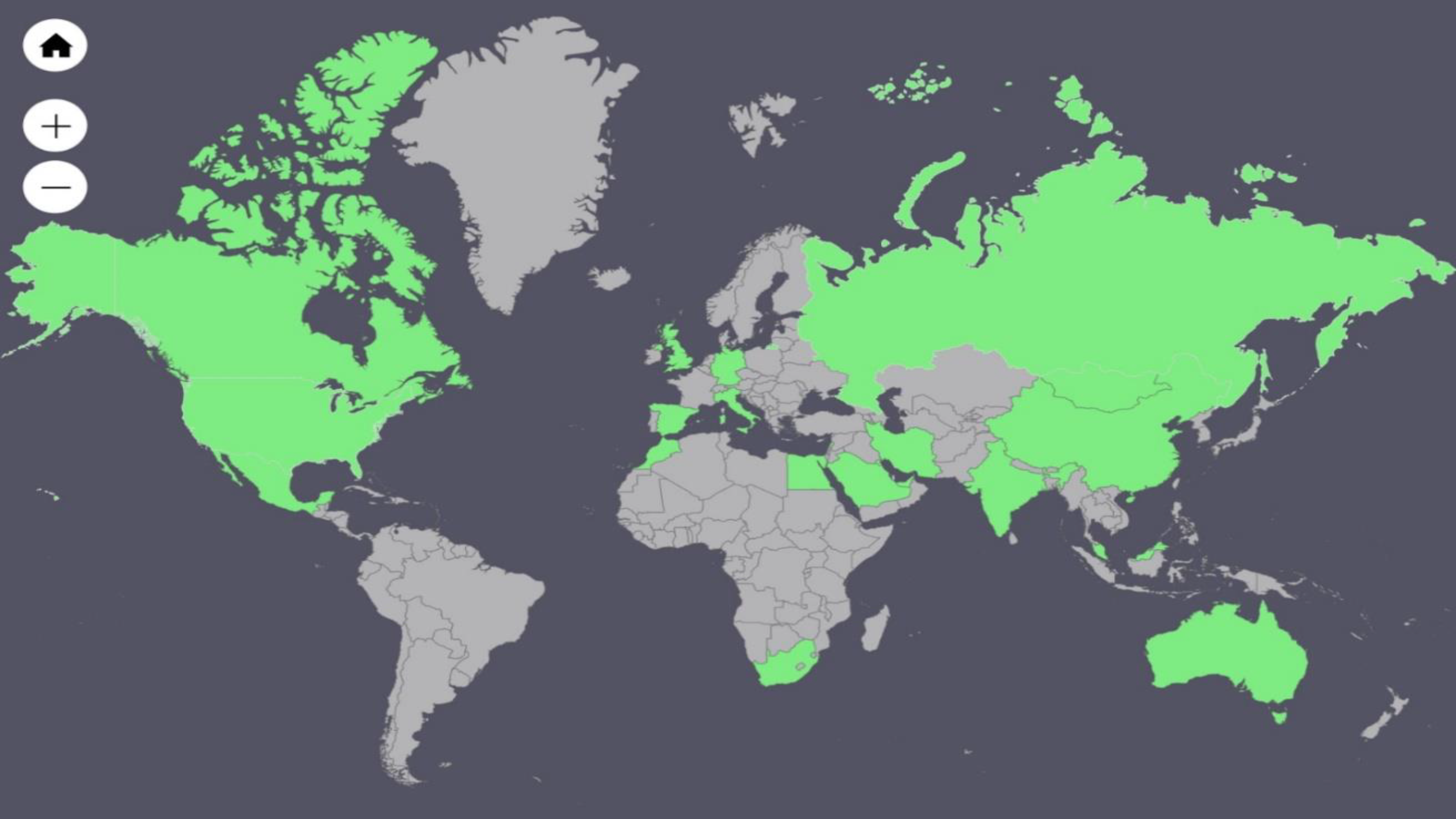

Supplement: Supplementary file 1 [file Image_1.TIFF]

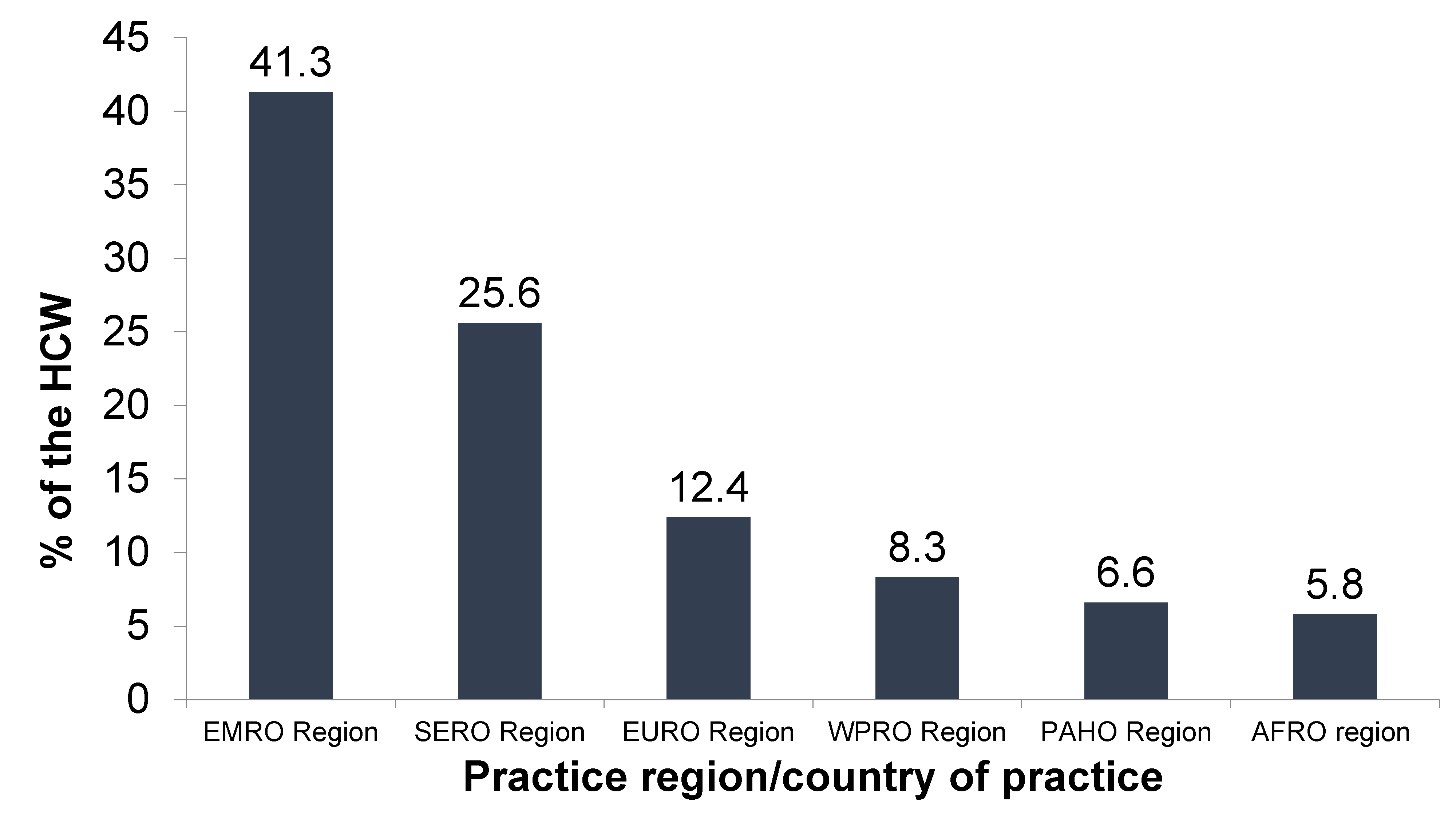

Supplement: Supplementary file 2 [file Image_2.TIFF]

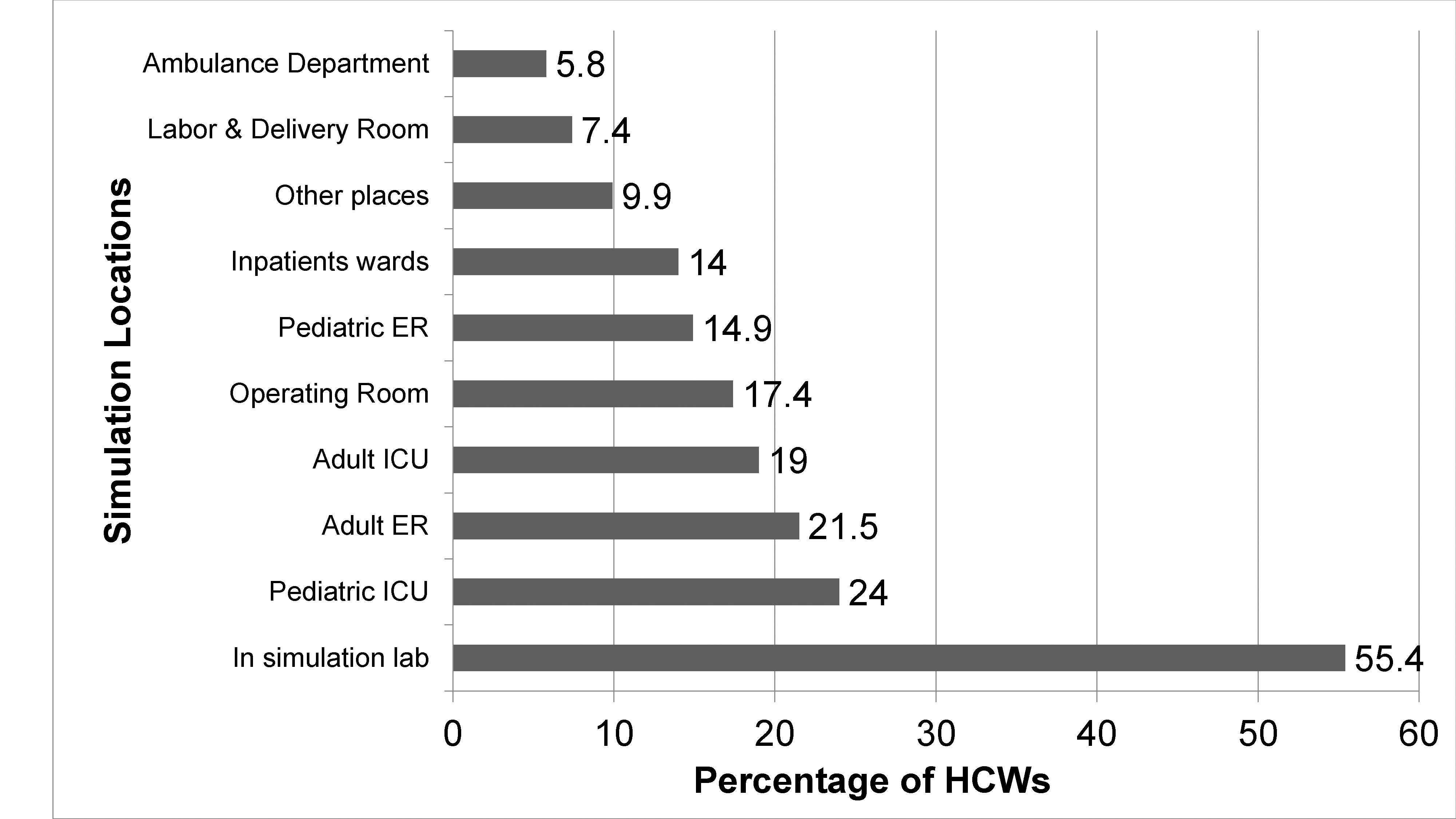

Supplement: Supplementary file 3 [file Image_3.TIFF]

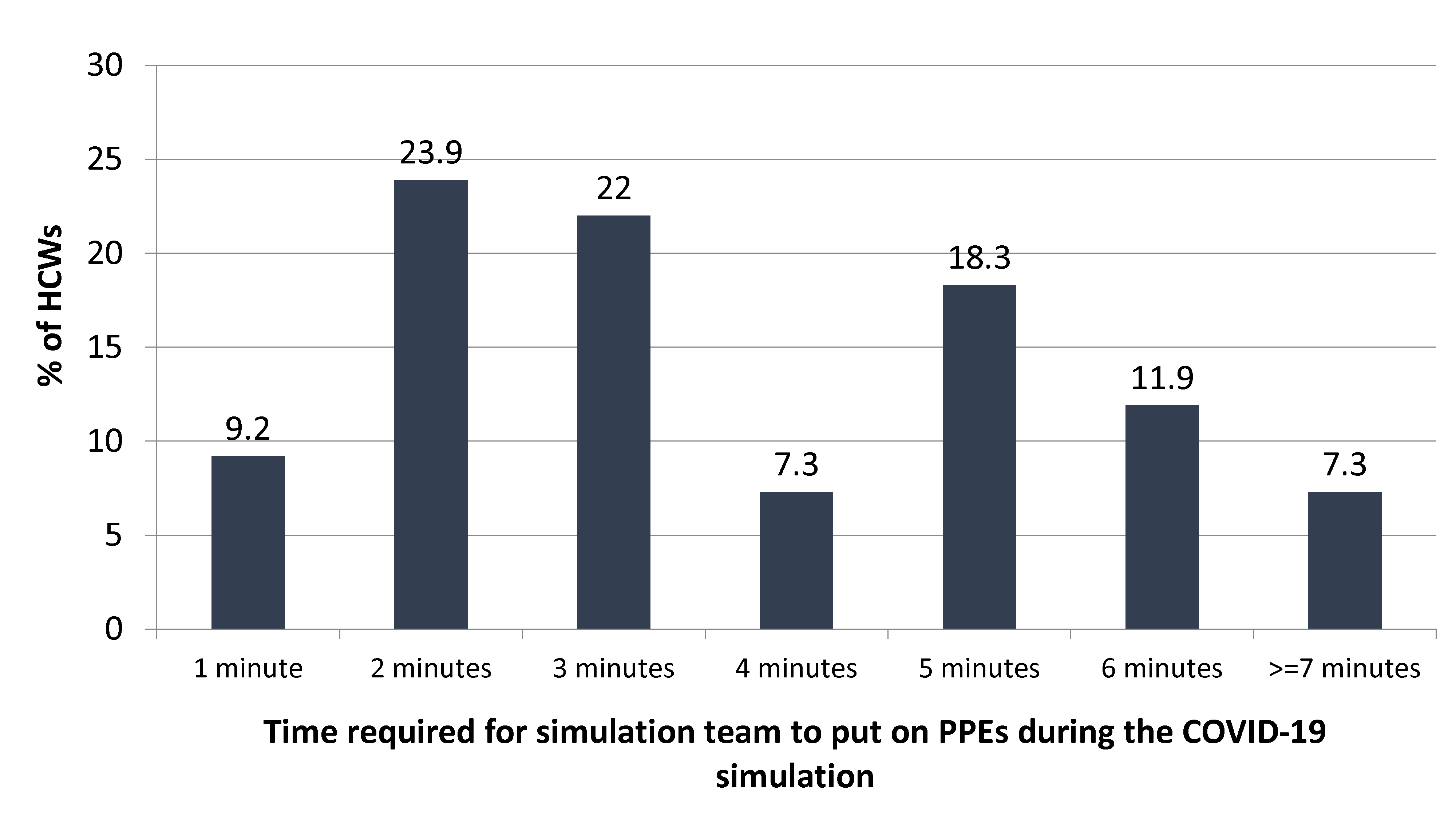

Supplement: Supplementary file 4 [file Image_4.TIFF]
